# Cardiovascular Risks Associated With Cannabis Use Among ED Patients

**DOI:** 10.7759/cureus.106400

**Published:** 2026-04-03

**Authors:** Jeffrey T Bowcutt, Brandon A Lopez, Lauren G Bothwell, Krishna K Paul, Diann Gaalema, Hani Jneid, Jeremy M Carter, Bryan Wilson, Olu Akinwande, Hailey P Hoffmann, Dietrich V Jehle

**Affiliations:** 1 Department of Emergency Medicine, University of Texas Medical Branch at Galveston, Galveston, USA; 2 Department of Emergency Medicine, University of Texas at Austin Dell Medical School, Austin, USA; 3 Department of Cardiology, University of Texas Medical Branch at Galveston, Galveston, USA

**Keywords:** alcohol related disorders, cannabis use disorder, cardiovascular disease, emergency medicine, marijuana

## Abstract

Objectives

Cannabis is one of the most widely used psychoactive substances, and many adults meet the criteria for Cannabis Use Disorder (CUD). Prior research on its association with cardiovascular disease (CVD) has yielded mixed results. This study evaluates the relationship between CUD and CVD among ED patients.

Methods

This retrospective, propensity-matched analysis examined records from 67 US healthcare organizations (HCOs) in the TriNetX database from 2005 to 2022. Adult ED patients with CUD documented within five years before or one month after an ED visit were compared with those without CUD. A secondary analysis compared patients with CUD to those with alcohol-related disorders (ARD). Outcomes included all-cause mortality, stroke, and myocardial infarction (MI) within three years of the ED visit. Matching controlled for demographics and comorbidities.

Results

A total of 1,748,263 ED patients were identified. After propensity matching, 598,114 adult patients remained, with 299,057 in each of the CUD and no-CUD groups. Patients with CUD experienced higher rates of mortality (RR 1.69, 95% CI 1.64-1.75) and MI (RR 1.07, 95% CI 1.02-1.12) within three years compared with those without CUD, with no significant difference in stroke (RR 0.96, 95% CI 0.90-1.02). In the secondary analysis, patients with CUD showed lower mortality but higher rates of stroke and MI than those with ARD.

Conclusion

Cannabis use was associated with increased risks of mortality and MI within three years of an ED visit. These findings add to the existing literature on the cardiovascular implications of cannabis use and may help inform health policy and clinical decision-making.

## Introduction

Cannabis, or marijuana, is one of the most widely used psychoactive substances in the world. Up to 147 million people, or approximately 2.5% of the world’s adult population, have reported using cannabis preparations [1]. Despite its popularity, the association between cannabis and adverse cardiovascular events is poorly understood. Prior meta-analyses examining this association have shown conflicting or inconclusive findings.

A 25-year prospective cohort study following 5,113 individuals, in which 84% of subjects (n=4286) self-reported marijuana use, concluded that neither cumulative lifetime use nor recent use of marijuana was associated with a higher incidence of cardiovascular disease (CVD) [2]. Additionally, a 2023 systematic review of 20 studies concluded that cannabis use was not associated with an increased risk of acute myocardial infarction (MI) or stroke [3]. On the other hand, other studies have linked marijuana use to an increased risk of acute adverse cardiovascular events. One large-scale systematic review identified 16 studies that found cannabis use to be a significant risk factor for MI or stroke among patients with a pre-existing cardiovascular event [4]. Another systematic review demonstrated an increased risk of hemorrhagic and ischemic stroke [5]. Each of these studies, regardless of outcome, had limitations such as inconsistency in defining marijuana dose, method of consumption (ie, smoking, ingestion, vaping), control for tobacco use, and frequency of use. As such, these studies support the need for further investigation.

This study aims to explore the relationship between cannabis use in ED patients and adverse cardiovascular events (ie, mortality, stroke, MI) and to provide a deeper understanding of the association between cannabis use and CVD.

This article was previously presented as a meeting abstract at the 2024 TCEP Connect Annual Meeting on April 20, 2024, and at the 2024 ACEP Scientific Assembly on September 30, 2024.

## Materials and methods

TriNetX is a large database that utilizes compiled, de-identified electronic medical records from numerous healthcare organizations (HCOs) in the US. The TriNetX platform integrates clinical data, including diagnoses, procedures, and laboratory results, and is linked to national death registries to enhance the accuracy of mortality data. The study utilized specific International Classification of Diseases (ICD), procedure, and laboratory codes to identify relevant diagnoses and outcomes (Appendix 1). These codes were applied consistently across the TriNetX dataset to ensure the accuracy and reliability of the data extraction process.

Cohort selection

This retrospective study utilized the US Collaborative Network, consisting of 67 HCOs and 113,383,545 patients. Our study population included adult patients who had ED visits recorded in the TriNetX database from January 17, 2005, to January 17, 2022. The primary cohort consisted of patients diagnosed with Cannabis Use Disorder (CUD), denoted by the ICD-10 code for “Cannabis Use, Unspecified, Uncomplicated,” within five years before or one month after their ED visit. A control group included patients who had a diagnosis of pharyngitis within five years before or one month after their ED visit and no diagnosis of CUD. The control group of patients without a diagnosis of CUD was too large for the database to perform propensity matching, so a diagnosis of pharyngitis was used to allow a more manageable comparison. To ensure a balanced comparison, propensity score matching was used to create comparable groups. Additionally, subgroup analysis was conducted comparing patients with CUD to those diagnosed with “Alcohol-Related Disorders” (ARD) but with no history of CUD.

Outcomes

The primary outcomes evaluated were all-cause mortality, stroke, and MI within three years following the ED visit in patients with CUD versus those without CUD. Patients with a history of these outcomes before the study period were excluded from the analysis. Propensity score matching was performed to adjust for potential confounders, ensuring balanced baseline characteristics between the cohorts. This matching was based on demographics and eight pre-existing medical conditions associated with cardiovascular mortality: nicotine dependence, ischemic heart disease, diabetes mellitus, overweight and obesity, acute kidney failure and chronic kidney disease, heart failure, cardiac arrest, and hypertensive diseases.

Secondary analysis

For the secondary analysis, we analyzed a cohort of patients with CUD without ARD versus patients with ARD without CUD. The outcomes evaluated remained the same (all-cause mortality, stroke, and MI). Propensity score matching was performed while controlling for demographics and the eight pre-existing medical conditions listed above.

Statistical analysis

A 1:1 propensity score matching was completed using linear regression for continuous variables and logistic regression for binary outcomes for each analysis. For the primary analysis, patients were matched for the following pre-existing diseases: diabetes mellitus (ICD-10: E08-E13), acute kidney failure and chronic kidney disease (ICD-10: N17-N19), overweight and obesity (ICD-10: E66), cardiac arrest (ICD-10: I46), ischemic heart diseases (ICD-10: I20-I25), heart failure (ICD-10: I50), hypertension (ICD-10: I10-I16), and nicotine dependence (ICD-10: F17.200). Greedy nearest-neighbor matching was utilized. Comparisons were made between cohorts before and after propensity matching in both the primary (CUD vs no CUD) and secondary (ARD vs CUD) analyses. Using the Measure of Association tool within the TriNetX platform, univariate analysis with chi-square and t-test was performed for each cohort and reported as descriptive statistics, risk ratios (RRs), 95% CIs for these ratios, and probability values (p-values).

The retrospective nature of this study involved the use of historical data from the TriNetX database, and all analyses were conducted using de-identified data. As this study involved secondary analysis of existing de-identified data, it was determined by the University of Texas Medical Branch Institutional Review Board (IRB) to be “not human subjects research” and thus did not require IRB review.

## Results

Patient characteristics

This study included 306,980 patients diagnosed with CUD and 1,441,283 patients diagnosed with pharyngitis (control). The mean age of the CUD cohort was 35.5 ± 13.9 years, compared to 36.9 ± 16.2 years in the control group, showing a statistically significant difference. Distribution by sex was also statistically different between the groups, with 125,771 (41.1%) females and 172,843 (56.4%) males in the CUD cohort, compared to 890,656 (64.8%) females and 456,799 (33.3%) males in the control cohort. All measured comorbidities varied significantly between the groups (Table 1).

After propensity score matching, the cohorts were balanced, with 299,057 patients in each group. The mean age remained statistically significantly different between the CUD group (35.4 ± 13.9 years) and the control group (35.6 ± 14.2 years). However, the sex distribution was almost identical post-matching, with females making up 125,701 (42.0%) of the CUD group and 125,536 (42.0%) of the control group, and males making up 165,795 (55.4%) of the CUD group compared to 165,949 (55.5%) of the control group. Race was relatively similar between the two cohorts. Post-matching, only ischemic heart disease, acute kidney failure and chronic kidney disease, heart failure, cardiac arrest, and nicotine dependence remained statistically significant. Despite this, all the standardized mean differences for the covariates were less than 0.1, signifying a well-balanced match (Table 1).

**Table 1:** Characteristics of adult emergency department patients diagnosed with CUD (n=306,980) versus those without CUD (n=1,441,283). * Cohort 1: CUD ** Cohort 2: No CUD CUD: Cannabis Use Disorder; Std diff.: Standardized difference; df: Degrees of freedom. P-values were calculated using the chi-square test for categorical variables and the t-test for continuous variables.

Cohort	Variable	Before Propensity Score Matching					After propensity score matching				
		Patients	% of cohort	P-value	Std. diff.	Chi^2^ / t-test	Patients	% of cohort	P-value	Std. diff.	Chi^2^ / t-test
1*	Age at index	306,241	100%	<0.001	0.096	t-test: t = -48.8, df ≈ 1.68 million	299,057	100%	<0.001	0.013	t-test: t = -5.51, df ≈ 598k
2**	Age at index	1,373,704	100%				299,057	100%			
1	Female	125,771	41.10%	<0.001	0.49	59192.17	125,701	42.00%	0.666	0.001	0.19
2	Female	890,656	64.80%				125,536	42.00%			
1	Black or African American	98,813	32.30%	<0.001	0.267	19415.62	92,825	31.00%	0.265	0.003	1.24
2	Black or African American	282,954	20.60%				92,426	30.90%			
1	Male	172,843	56.40%	<0.001	0.479	57456.11	165,795	55.40%	0.689	0.001	0.16
2	Male	456,799	33.30%				165,949	55.50%			
1	White	155,867	50.90%	<0.001	0.161	6534.79	155,037	51.80%	0.041	0.005	4.17
2	White	808,896	58.90%				155,826	52.10%			
1	American Indian or Alaska Native	1,357	0.40%	<0.001	0.013	42.27	1,303	0.40%	0.769	0.001	0.09
2	American Indian or Alaska Native	4,992	0.40%				1,318	0.40%			
1	Unknown race	31,901	10.40%	<0.001	0.01	27.03	31,655	10.60%	0.357	0.002	0.85
2	Unknown race	147,506	10.70%				31,436	10.50%			
1	Native Hawaiian or Other Pacific Islander	2,204	0.70%	<0.001	0.059	739.13	2,203	0.70%	<0.001	0.009	12.87
2	Native Hawaiian or Other Pacific Islander	18,027	1.30%				1,972	0.70%			
1	Unknown gender	7,627	2.50%	<0.001	0.04	425.91	7,561	2.50%	0.928	<0.001	0.01
2	Unknown gender	26,249	1.90%				7,572	2.50%			
1	Hispanic or Latino	25,503	8.30%	<0.001	0.074	1295.13	25,325	8.50%	0.781	0.001	0.08
2	Hispanic or Latino	144,167	10.50%				25,385	8.50%			
1	Not Hispanic or Latino	207,997	67.90%	<0.001	0.038	357.9	202,117	67.60%	0.687	0.001	0.16
2	Not Hispanic or Latino	908,492	66.10%				202,263	67.60%			
1	Other race	12,296	4.00%	<0.001	0.027	169.94	12,231	4.10%	0.349	0.002	0.88
2	Other race	62,539	4.60%				12,375	4.10%			
1	Asian	3,803	1.20%	<0.001	0.151	4405.76	3,803	1.30%	0.25	0.003	1.32
2	Asian	48,790	3.60%				3,704	1.20%			
1	Ischemic heart diseases	12,788	4.20%	<0.001	0.031	253.52	12,046	4.00%	<0.001	0.012	21.24
2	Ischemic heart diseases	49,128	3.60%				11,355	3.80%			
1	Diabetes mellitus	25,120	8.20%	<0.001	0.008	14.84	24,048	8.00%	0.696	0.001	0.15
2	Diabetes mellitus	115,610	8.40%				23,966	8.00%			
1	Overweight and obesity	36,791	12.00%	<0.001	0.116	3141.39	36,326	12.10%	0.127	0.004	2.32
2	Overweight and obesity	220,438	16.00%				36,712	12.30%			
1	Acute kidney failure and chronic kidney disease	20,960	6.80%	<0.001	0.15	7003.06	18,248	6.10%	<0.001	0.015	34.16
2	Acute kidney failure and chronic kidney disease	48,336	3.50%				17,181	5.70%			
1	Heart failure	8,271	2.70%	<0.001	0.063	1150.4	7,549	2.50%	<0.001	0.016	40.28
2	Heart failure	24,269	1.80%				6,798	2.30%			
1	Cardiac arrest	539	0.20%	<0.001	0.032	367.39	449	0.20%	<0.001	0.012	21.31
2	Cardiac arrest	886	0.10%				321	0.10%			
1	Hypertensive diseases	61,383	20.00%	<0.001	0.016	66.39	58,481	19.60%	0.595	0.001	0.28
2	Hypertensive diseases	266,481	19.40%				58,318	19.50%			
1	Nicotine dependence, unspecified, uncomplicated	77,972	25.50%	<0.001	0.447	64498.3	70,798	23.70%	0.001	0.009	12
2	Nicotine dependence, unspecified, uncomplicated	123,342	9.00%				71,940	24.10%			

Primary analysis results

When comparing outcomes before propensity score matching, the mortality rate was significantly higher in the CUD group, with 9,107 (2.98%) deaths compared to 19,627 (1.43%) deaths in the control group (RR 2.08, 95% CI: 2.03-2.13, p < 0.001). After matching, the mortality rate in the CUD group was 8,690 (2.91%) versus 5,131 (1.72%) in the control group and remained statistically significant, with an RR of 1.69 (95% CI: 1.64-1.75, p < 0.001). Stroke incidence did not differ significantly before matching (0.67% vs. 0.70%, RR 0.96, 95% CI: 0.91-1.01, p = 0.08) or after matching, with 1,959 patients (0.66%) in the CUD group experiencing stroke compared to 2,037 (0.68%) in the control group (RR 0.96, 95% CI: 0.90-1.02, p = 0.21). MI rates were higher in the CUD group before matching (0.95% vs. 0.73%, RR 1.31, 95% CI: 1.25-1.36, p < 0.001) as well as after matching (0.91% vs. 0.85%, RR 1.07, 95% CI: 1.02-1.13, p = 0.01) (Table 2).

**Table 2:** Mortality, stroke, and MI outcomes for patients with CUD versus those without CUD before and after propensity score matching. CUD: Cannabis Use Disorder; RR: Relative risk; MI: Myocardial infarction. P-values were calculated using the chi-square test.

Outcomes	Before propensity score matching					After propensity score matching				
	CUD (%)	No CUD (%)	RR (95% CI)	Chi^2^	P-value	CUD (%)	No CUD (%)	RR (95% CI)	Chi^2^	P-value
Deceased	9,107 (2.98%)	19,627 (1.43%)	2.08 (2.03, 2.13)	3,556.73	<0.001	8,690 (2.91%)	5,131 (1.72%)	1.69 (1.64, 1.75)	938.09	<0.001
Stroke	2,044 (0.67%)	9,566 (0.70%)	0.96 (0.91, 1.01)	3.13	0.08	1,959 (0.66%)	2,037 (0.68%)	0.96 (0.90, 1.02)	1.57	0.21
MI	2,898 (0.95%)	9,943 (0.73%)	1.31 (1.25, 1.36)	163.18	<0.001	2,722 (0.91%)	2,533 (0.85%)	1.07 (1.02, 1.13)	6.77	0.01

Secondary analysis results

Comparing CUD to ARD post-propensity matching, the CUD group had significantly higher rates of stroke (0.56% vs. 0.43%, RR 1.30, 95% CI: 1.20-1.42, p < 0.001) and MI (0.79% vs. 0.48%, RR 1.63, 95% CI: 1.51-1.76, p < 0.001); however, mortality was higher in the ARD group (2.43% vs. 3.32%, RR 0.73, 95% CI: 0.71-0.76, p < 0.001) (Table 3).

**Table 3:** Mortality, stroke, and MI outcomes for patients with CUD versus ARD before and after propensity score matching. CUD: Cannabis Use Disorder; ARD: Alcohol-Related Disorders; RR: Relative risk; MI: Myocardial infarction. P-values were calculated using the chi-square test.

Outcomes	Before propensity score matching					After propensity score matching:				
	CUD (%)	ARD (%)	RR (95% CI)	Chi^2^	P-value	CUD (%)	ARD (%)	RR (95% CI)	Chi^2^	P-value
Deceased	5,556 (2.38%)	42,766 (4.99%)	0.48 (0.46, 0.49)	2960.73	<0.001	5,415 (2.43%)	7,384 (3.32%)	0.73 (0.71, 0.76)	313.68	<0.001
Stroke	1,291 (0.55%)	5,056 (0.59%)	0.94 (0.88, 1.00)	4.32	0.04	1,254 (0.56%)	963 (0.43%)	1.30 (1.20, 1.42)	38.32	<0.001
MI	1,816 (0.78%)	5,358 (0.63%)	1.25 (1.18, 1.31)	65.78	<0.001	1,753 (0.79%)	1,075 (0.48%)	1.63 (1.51, 1.76)	163.59	<0.001

## Discussion

This retrospective study examined the association between CUD and adverse cardiovascular outcomes among patients visiting the ED. Although cannabis is often perceived as relatively harmless [6], our findings before and after propensity matching suggest that patients with CUD may have a higher risk of mortality and MI compared to controls. However, stroke incidence did not differ significantly between the groups. Additionally, when comparing CUD to ARD, the CUD cohort demonstrated higher rates of stroke and MI after matching, though overall mortality remained higher in the ARD group.

The relationship between CUD and all-cause mortality remains controversial in the literature. Sun Y et al. reported an elevated risk ratio for CVD mortality among cannabis users but did not find a significant increase in overall mortality. Interestingly, early initiation of cannabis use (before age 18) was associated with a higher risk of CVD mortality, suggesting that duration of exposure may be a critical factor [7]. Vallée A similarly found increased CVD mortality in females but no difference in all-cause mortality for either sex [8]. Conversely, studies by Vozoris NT et al., Calabria B et al., and the Institute of Medicine concluded that cannabis use was not associated with an increase in overall mortality [9-11]. These discrepancies likely stem from differences in study populations, patterns of cannabis use, and unmeasured confounding variables, highlighting the need for further investigation into the long-term effects of CUD.

These findings regarding stroke incidence in patients with CUD contrast with some of the existing literature. Although this analysis did not find a statistically significant difference in stroke risk among patients with CUD, a large meta-analysis of over three million individuals reported a small increase in the odds of stroke (OR 1.17, 95% CI 1.10-1.25), with a mean stroke onset age of just 26.2 years [5]. This early age of onset is consistent with other reports [12], raising concerns about early cardiovascular compromise in younger users. Some studies also suggest a temporal relationship between recent cannabis use and stroke onset [12]. Notably, San Luis CV et al. reported a decreased risk of stroke among cannabis users [13], while Desai R et al. found that geriatric patients with both CUD and peripheral vascular disease had a significantly increased stroke risk compared to controls [14]. These divergent findings emphasize the complexity of the cannabis-stroke relationship and the possible influence of age, comorbidities, and timing of exposure.

This study demonstrated that cannabis use may increase MI risk. We found that post-propensity-matched patients with CUD had significantly higher MI rates compared to controls. This aligns with findings from Desai R et al., who reported elevated MI risk among cannabis users (OR 1.08, 95% CI 1.04-1.12) [14], and with other studies indicating an increased risk of acute MI within one hour of cannabis use [15]. However, some reports have failed to find a significant association between cannabis use and cardiovascular outcomes after adjusting for sociodemographic and behavioral factors [16]. Importantly, frequent cannabis use following MI was associated with increased mortality in a study by Mukamal KJ et al., suggesting that cannabis may worsen outcomes in patients with pre-existing heart disease [17]. The heterogeneity in findings across studies may be attributed to variations in study design, sample size, and the potency or form of the drug.

When comparing CUD and ARD, this study found that patients with CUD had significantly higher rates of MI and stroke. While alcohol use is well known to increase cardiovascular risks, especially at high doses, there is also conflicting evidence suggesting a dose-dependent protective effect of moderate alcohol consumption on ischemic heart disease [18]. Alcohol has also been linked to dysrhythmias and cardiomyopathy, both of which can elevate stroke risk [19]. This study did not assess consumption patterns for either substance, which may explain the divergence from prior findings on alcohol’s nuanced cardiovascular impact. Nevertheless, growing literature suggests that cannabis may also exhibit dose-dependent cardiovascular effects, similar to alcohol.

A recent 2024 study from Amsterdam highlighted the role of consumption methods in determining cardiovascular risk. Smoking cannabis was found to elevate MI risk, particularly in younger adults, likely due to combustion-related toxins such as carbon monoxide [20]. In contrast, alternative forms of intake (such as edibles or vaping) may pose fewer cardiovascular risks. This finding reinforces the need to evaluate not only cannabis use but also the mode and frequency of consumption when assessing cardiovascular outcomes.

Limitations

This study has several limitations that must be acknowledged. As a retrospective study, our analysis cannot establish causation between cannabis use and adverse cardiovascular events. The observational nature of the study limits our ability to draw definitive conclusions about causality. While we utilized propensity score matching to control for confounding variables, there may still be unmeasured confounders that could bias our results, even in a well-matched cohort. For instance, lifestyle factors, socioeconomic status, and genetic predispositions, which were not accounted for, might influence both cannabis use and cardiovascular outcomes. Additionally, due to the privacy policies of the TriNetX database, we were unable to evaluate site clustering or specific hospital distributions. This restriction may limit the generalizability of our findings, as the impact of cannabis use on cardiovascular events could vary by location and healthcare setting.

The study relies on ICD codes to identify CUD, which may not capture the full extent of use, including dosage, frequency, and method of consumption (e.g., smoking, ingestion, vaping). Some patients who use cannabis but were not identified as having a disorder may therefore have been missed. These factors may provide additional context for understanding the nuanced effects of cannabis on cardiovascular health; however, this information is not available within the database. In addition, using nicotine dependence as a covariate may miss some individuals who smoke cigarettes. Moreover, although the TriNetX platform integrates data from various HCOs, there is potential for incomplete data, particularly regarding mortality events occurring outside affiliated HCOs. Although 94% of HCOs in the network are linked to US death registries, there remains a small risk of missed death events. There is also significant variability in the composition of cannabis products, including differences in the concentrations of tetrahydrocannabinol and cannabidiol, which could have differing effects on cardiovascular health. This heterogeneity was not available for capture in our study. Additionally, our study utilized historical data from 2005 to 2022, during which time the legal status of and societal attitudes toward cannabis changed significantly [8]. These changes may have affected patterns of use and reporting accuracy over time.

## Conclusions

This large, real-world analysis reveals a concerning association between CUD and adverse cardiovascular events. Following propensity score matching, CUD was associated with increased risks of mortality and MI, though no significant difference was found in stroke incidence. These findings suggest that CUD may be an underrecognized cardiovascular risk factor, even in the absence of detailed consumption data. With cannabis becoming increasingly legal and socially accepted, clinicians should screen for CUD and consider cardiovascular risk stratification as part of routine care for patients with cannabis use. Furthermore, public health policies that promote cannabis as a harmless recreational substance may need to be reevaluated in light of emerging data suggesting potential harms. Comparative studies examining the long-term effects of cannabis, alcohol, and cigarette or tobacco smoking will be critical to shaping evidence-based clinical guidelines.

## Appendices

Appendix 1

Greedy Nearest Neighbor Matching (NNM)

The most common implementation of propensity score matching is pair matching, in which pairs of treated and control participants are formed. There are several common implementations of pair matching. The most commonly used is greedy NNM, which we used, in which a treated participant is selected at random and then matched to the control participant whose propensity score is closest to that of the treated participant. The process is described as greedy because, at each stage, the control participant who is closest to the currently considered treated participant is selected, even if that untreated participant would serve better as a control for a subsequent treated participant. This process is then repeated until a matched control participant has been selected for each treated participant. This process generally uses matching without replacement, so that once a control participant is matched to a treated participant, that control participant is no longer available for matching to a subsequent treated participant.

A refinement of NNM is NNM with a caliper restriction. Using this approach, a control participant is considered an acceptable match for a treated participant only if the difference in their propensity scores is less than a maximum amount (the caliper width or distance). For technical reasons, one typically matches on the logit of the propensity score and uses a caliper width defined as a proportion (0.1-0.2) of the SD of the logit of the propensity score. A crucial step in any study that uses propensity score matching is to assess the degree to which matching on the propensity score resulted in the formation of a matched sample in which the distribution of baseline characteristics is similar between treated and control participants. This assessment is critical, as it allows both the researcher and the readers to determine whether matching on the estimated propensity score has removed systematic baseline differences between treatment groups. The standardized difference, which is the difference in means expressed in units of SD, is often used to assess the similarity of matched treated and control participants. Some authors have suggested that a threshold of 0.10 (or 10%) be used to denote acceptable balance after matching. Once acceptable balance has been achieved, analysts can unblind themselves to the outcome and compare outcomes between treated and control participants in the matched sample. The analyses conducted in the propensity score-matched sample can be similar to those that would be performed in an RCT with a similar outcome.
